# State-of-the-art management of nasopharyngeal carcinoma: current and future directions

**DOI:** 10.1038/sj.bjc.6602449

**Published:** 2005-03-01

**Authors:** M Agulnik, L L Siu

**Affiliations:** 1 Department of Medical Oncology and Hematology, Princess Margaret Hospital, University Health Network, 610 University Avenue, Toronto, Ontario, Canada M5G 2M9

**Keywords:** nasopharyngeal carcinoma, chemotherapy, radiotherapy, molecular targeted agents, immunotherapy

## Abstract

Nasopharyngeal carcinoma (NPC) is a distinct type of head and neck cancer. Approximately 70% of patients with newly diagnosed NPC present with locally advanced disease. Phase III clinical trials support the addition of chemotherapy to radiotherapy for the initial treatment of these patients. Once metastatic disease develops, practices become varied. Further experience needs to be gained with both targeted therapies and immunotherapy to gauge whether they will improve treatment outcomes in NPC.

Nasopharyngeal carcinoma (NPC) is a distinct type of head and neck cancer that differs from other malignancies of the upper aerodigestive tract with respect to epidemiology, pathology, clinical presentation and responses to treatment. Outside of endemic areas such as Southeast Asia, this type of tumour is extremely rare with an incidence of less than 1/100 000 (Fandi *et al*, 1994). Patients with stage I and II disease have a high rate of cure with radiotherapy (RT) alone, while the prognosis for those with distant metastatic spread remains poor. Approximately 70% of patients with newly diagnosed NPC present with locally advanced, nonmetastatic stage III or IV disease. For this patient population, surgery has a limited role and RT delivered in combination with chemotherapy has become the standard of care. However, the timing, dosing, duration and optimal regimens of chemotherapy drugs to be combined with RT remain controversial. Several randomised trials have evaluated neoadjuvant, concurrent and adjuvant chemotherapy in different combinations with RT. This review focuses on the evidence to support treatment of locally advanced NPC, and provides an update of promising therapies that are on the horizon.

## THE ADDITION OF CHEMOTHERAPY TO RT FOR LOCALLY ADVANCED NPC

A Medline and American Society of Clinical Oncology abstract search was conducted using the keywords ‘nasopharyngeal cancer/carcinoma’ and ‘radiotherapy/radiation therapy’ and ‘chemotherapy’. Articles and abstracts were eligible for review if patients were randomly assigned to receive chemotherapy and RT or RT alone. There are 11 published papers and four abstracts, which present the results of 13 randomised clinical trials comparing chemotherapy with RT to RT alone in patients with locally advanced NPC (Tables 1 and 2). Only studies published in the English literature were included in this review.

### Neoadjuvant chemotherapy

Four trials have assessed the role of neoadjuvant chemotherapy followed by RT *vs* RT alone (VUMCA 1996; Chua *et al*, 1998; Ma *et al*, 2001; Hareyama *et al*, 2002). The VUMCA I trial treated 339 patients in the neoadjuvant therapy arm with cisplatin, epirubicin and bleomycin. After a median follow-up duration of 49 months, this trial failed to show an overall survival (OS) advantage in the neoadjuvant therapy arm but did report a statistically significant increase in 3-year disease-free survival (DFS) rates (52 *vs* 32%, interpolated). However, a treatment-related toxic death rate of 8% was encountered in the neoadjuvant therapy arm in this multicentre trial. In a small trial, Hareyama *et al* randomised 80 patients to two cycles of cisplatin and 5-fluorouracil administered prior to RT *vs* RT alone. Trends towards improved 5-year DFS and OS were demonstrated in the neoadjuvant therapy arm, but statistically significant differences were not achieved after a median follow-up period of 49 months. In their initial reporting after a median follow-up duration of 30 months, Chua *et al* showed similar results with their neoadjuvant combination of cisplatin and epirubicin. Again, trends towards improved OS and DFS were seen in the neoadjuvant therapy arm, but the results were not statistically significant. Lastly, Ma *et al* compared two to three cycles of bleomycin, cisplatin and 5-fluorouracil followed by RT to RT alone and showed a statistically significant prolongation of DFS in the chemotherapy group (59 *vs* 49% at 5 years). A trend towards OS was observed in the neoadjuvant arm of the trial. Updated combined data from these two latter trials (Chua DTT *et al*, 2004) on 784 patients, after a median follow-up period of 67 months, reported a statistically significant improvement in 5-year DFS (51 *vs* 43%) favouring the neoadjuvant therapy arm, but not in 5-year OS (62 *vs* 58%). Reductions in both locoregional and distant failures were observed. Although it has been suggested by the trends, to date, no statistically significant OS advantage has been documented in a phase III randomised trial using neoadjuvant chemotherapy followed by RT.

### Concurrent chemotherapy

Two trials have compared concurrent chemotherapy and RT *vs* RT alone (Chan *et al*, 2002; Lin *et al*, 2003). Lin *et al* randomised patients to concurrent chemoradiotherpy *vs* RT alone. Using the 1992 American Joint Committee on Cancer (AJCC) staging system, all 284 patients had either stage III or IV disease. Patients randomised to the concurrent chemotherapy arm of the study received two cycles of cisplatin mixed with 5-fluorouracil administered as a 96-h continuous infusion during weeks 1 and 5 of RT. The 5-year OS rates for the chemotherapy arm were 72% compared with 54% in the control arm; the 5-year DFS rates were 72 *vs* 53%, respectively. Both comparisons were statistically significant. In a similar study, Chan *et al* randomised patients to adjuvant weekly low-dose cisplatin and standard RT *vs* RT alone. Over 90% of their patients were Ho tumour stage III or IV. The 2-year DFS in the chemotherapy arm was 76% compared to 69% in the RT alone group, which did not achieve statistical significance, and OS was not reported in the initial analysis after a follow-up period of 33 months. Updated data from the Chan *et al* (2004) study, after a median follow-up duration of 65 months, reported 5-year DFS of 60 *vs* 52% and 5-year OS of 70 *vs* 59%, both reaching borderline statistical significance (*P*=0.06 and 0.05, respectively), in favour of the concurrent chemoradiotherapy arm. Reclassification of the 350 patients on this trial using the 1997 AJCC staging system revealed that over 70% were of stage III and IV. Subgroup analysis demonstrated that patients with T3 and T4 disease derived the most benefit. Based on these two randomised trials, patients with advanced locoregional NPC benefit from concurrent chemoradiotherapy over RT alone.

### Adjuvant chemotherapy

The first published randomised trial in locoregional NPC compared adjuvant chemotherapy after RT to RT alone (Rossi *et al*, 1988). The regimen used in this 4-year multicentre randomised trial was vincristine, cyclophosphamide and adriamycin. Patients were treated with 6 monthly cycles of treatment following their RT. Analysis of the patient data at 4 years failed to show a statistically significant change in DFS or OS between the two groups. As well, interestingly, the pattern of failure in both groups was similar with distant metastasis accounting for 50% of relapse failures. An important factor to consider is that the most active agent in NPC, cisplatin, has been omitted from their regimen. A second adjuvant trial did incorporate cisplatin with 5-fluorouracil and leucovorin postradiation in patients with locally advanced NPC (Chi *et al*, 2002). The chemotherapy delivered in the adjuvant arm consisted of the three agents given as a 24-h infusion, for 9 weekly cycles. At 5 years, there was no noted statistically different OS or DFS between the two groups of patients. Based on these two trials, adjuvant chemotherapy alone cannot be recommended for patients with advanced NPC.

### Neoadjuvant and adjuvant chemotherapy

Only one phase III trial of 77 patients has evaluated the combined role of neoadjuvant and adjuvant chemotherapy (Chan *et al*, 1995). Neoadjuvant and adjuvant chemotherapy consisted of two and four cycles of cisplatin and 5-fluorouracil, respectively. After a follow-up duration of 28.5 months, no significant difference has been observed in either the DFS or OS.

### Concomitant and adjuvant chemotherapy

A major breakthrough in the management of locally advanced NPC came about in 1998 with the publication of the phase III randomised Intergroup study 0099 (Al-Sarraf *et al*, 1998). Both arms of the study received identical RT, while the chemotherapy arm of the study received both concomitant and adjuvant chemotherapy. Cisplatin as a single agent was administered on days 1, 22 and 43 of the concurrent RT, and three adjuvant cycles of cisplatin with 5-fluorouracil were given monthly after completion of chemoradiotherapy. At 3 years, the DFS was 69% in the chemotherapy group and 24% in the RT alone arm. Their survival rates at 3 years were 78 *vs* 47%, favouring chemotherapy. Updated analysis at 5 years (Al-Sarraf *et al*, 2001) confirms the benefit of treatment with 5-year DFS rates of 58 *vs* 29% and 5-year OS rates of 67 *vs* 37%, both favouring the combined therapy arm. The National Cancer database has been analysed for changing patterns of practice since the first published results of the Intergroup 0099 study data in 1998. Of all patients enrolled in the database and matching the eligibility criteria of the Intergroup 0099 study, only 38% received chemotherapy along with RT prior to 1997, while since the publication of the data, 65% of these patients have received concurrent and adjuvant chemotherapy (Hoffman *et al*, 2004).

One-quarter of all patients treated on the Intergroup 0099 protocol had World Health Organization (WHO) stage I histology (keratinising squamous cell carcinoma). A phase III randomised trial using a similar chemotherapy and RT plan has been completed, with enrolment restricted to patients with WHO type IIa (nonkeratinising squamous cell carcinoma) and IIb (undifferentiated carcinoma) histologies (Wee *et al*, 2004). The chemotherapy regimen differed slightly from the Intergroup 0099 trial with the dose of cisplatin given in divided doses rather than one dose, but the total dose remained the same. The 2-year DFS and OS rates were statistically significant and favoured the use of chemotherapy. The Hong Kong NPC Study Group (Lee *et al*, 2004) randomised patients with nonkeratinising or undifferentiated NPC to the identical Intergroup 0099 regimen. On preliminary analysis after a median follow-up period of 2.3 years, the trends favoured the addition of chemotherapy with respect to DFS, but no benefit in OS was noted. Locoregional control was improved with the addition of chemotherapy.

In addition to the three trials mentioned above, which compared concurrent chemoradiotherapy plus adjuvant chemotherapy *vs* RT alone, a factorial study of four different regimens has been published (Kwong *et al*, 2004). This study assessed the combination of RT alone *vs* three other schemas: RT with adjuvant chemotherapy, concurrent chemoradiotherapy and lastly, concurrent chemoradiotherapy followed by adjuvant chemotherapy. UFT (uracil and tegafur in 4 : 1 molar ratio) was used as the concurrent chemotherapy agent, while the adjuvant chemotherapy protocol consisted of alternating cycles of cisplatin/5-fluorouracil with vincristine/bleomycin/methotrexate. Although a trend towards improved DFS and OS was noted with the addition of concurrent chemotherapy, it did not reach statistical significance at 3 years. In assessing distant metastases rates, a significant reduction was attributable to concurrent chemotherapy. In this study, adjuvant chemotherapy did not improve outcome.

## SUMMARY OF EVIDENCE: THE ADDITION OF CHEMOTHERAPY TO RT FOR LOCALLY ADVANCED NPC

Based on published randomised trials that addressed the value of adding chemotherapy to RT in locally advanced NPC, several conclusions may be drawn. It is important to note that these trials were completed over a 16-year time span (1988–2004) in different geographic areas. As such, several tumour staging systems and improved diagnostic techniques have been utilised, which might have contributed to a phenomenon of stage migration (Feinstein *et al*, 1985). That withstanding, the timing of chemotherapy appears to impact on clinical outcome. Concurrent chemotherapy delivered with RT has consistently produced a survival benefit when compared to RT alone, achieving 5-year OS rates of about 70% with combined modality therapy in patients with nonmetastatic stage III and IV disease.

The addition of further chemotherapy to concurrent chemoradiotherapy, delivered in a neoadjuvant or adjuvant sequence, might further augment disease control. Reductions in both locoregional and distant failures have been reported with the addition of chemotherapy to RT, when delivered using validated regimens in adequate dosages. In reviewing the 13 randomised trials, chemotherapy compliance rates differed dramatically depending on the timing of therapy. In the neoadjuvant setting, 87–100% of patients received the prescribed cycles of chemotherapy, while 44–93% of patients scheduled for concurrent chemotherapy received their planned cycles. Even fewer, 14–55% of patients, completed their planned adjuvant chemotherapy. Clearly, chemotherapy dose intensity is most optimally maintained in the induction setting. This disparity in dose intensities may partially explain the lack of benefit associated with the administration of adjuvant chemotherapy alone. Treatment strategies to optimise chemotherapy delivery to achieve locoregional and distant control should utilise this information and consider the combination of induction chemotherapy followed by concurrent therapy. Incorporation of newer, less toxic and more effective anticancer agents such as gemcitabine, the taxanes or molecular targeted agents into combined modality regimens warrant continual exploration in locally advanced NPC. Collection of accurate toxicity data, especially those related to nutritional status, salivary and swallowing functions, ototoxicity, neuropathy and other late side effects, is vital to the planning of appropriate remedies.

## RT TECHNIQUES FOR LOCALLY ADVANCED NPC

Tumour control of NPC has been highly correlated with the RT dose delivered to the tumour. In the above-mentioned randomised trials, RT dosing is summarised in Table 3. The dosing in all studies is similar and most are using a split field technique with two lateral opposed facial fields and an anterior field if necessary. A few studies enrolled patients and treated them with hyperfractionated RT twice per day, but the majority of patients in these studies still received conventional fractionation. Only one study included intracavitary brachytherapy for patients with persistent local disease (Chan *et al*, 2002).

In order to achieve tumour control for carcinoma of the nasopharynx, dosing of greater than 67 Gray (Gy) is required; however, better technical accuracy of RT delivery can also improve upon disease control. In 1998, the use of three–dimensional (3D) intensity-modulated RT (IMRT) for NPC was implemented at the Memorial Sloan-Kettering Cancer Centre. Initial treatment reported an increased target dose delivery from 67.0 to 77.3 Gy with IMRT as compared to conventional treatment methods. Dose to normal structures such as the spinal cord, mandible, temporal lobes and parotid glands all decreased with IMRT (Hunt *et al*, 2001). A 2-year follow-up of the initial 39 patients treated with IMRT confirms a local relapse-free survival (LRFS) of 97 *vs* 78% in historical controls treated with conventional technique with a 3D boost, suggesting an improved locoregional control with IMRT (Wolden *et al*, 2002).

The University of California – San Francisco has also extensively studied the use of IMRT in the treatment of NPC (Sultanem *et al*, 2000; Xia *et al*, 2000; Lee *et al*, 2002). Initially, IMRT treatment plans were compared with conventional treatment plans for a case of locally advanced NPC. Their planning confirmed that IMRT techniques improved tumour target coverage and spared sensitive normal tissue structures. Subsequently, 35 patients were treated with IMRT for NPC. In all, 72% of these patients had 1997 AJCC stage III or IV disease. At 22 months of follow-up, no local or regional failures had been documented. This local control was attributable to improved tumour target coverage, increased total dose and increased dose per fraction to the tumour target. The mean dose delivered to the tumour target was 75.8 Gy. Follow-up at 31 months with a total of 67 patients confirmed the above and reported only one local recurrence at the primary site. The vast majority of failures in this series were attributed to distant metastases. Of note, in this series, 75% of the patients did receive concurrent and adjuvant chemotherapy as per the Intergroup protocol 0099 regimen. The Prince of Wales Hospital (PWH) experience in Hong Kong further confirms the benefits of IMRT. In treating 63 newly diagnosed patients with NPC (stage I–IV disease), a 3-year LRFS of 92% and OS of 90% are observed (Kam *et al*, 2004).

Aside from documenting improved LRFS and OS with IMRT, both the UCSF and PWH experiences confirm favourable toxicity profiles with IMRT. In spite of the increased dose, no increase in complication was reported, likely attributable to the reduced volumes to normal structures irradiated. Xerostomia is the most common side effect experienced by patients treated with conventional two-dimensional (2D) RT for NPC. Up to 97% of treated patients have reported xerostomia as a complication of therapy (Talmi *et al*, 2002). The UCSF data report no chronic xerostomia at 2 years, while the PWH experience reports 23% grade 2–3 xerostomia at 2 years with further decrease to 17% at 2 years in the subset of patients treated with less than 31 Gy to the parotid gland.

Although large randomised studies are not available to confirm the advantage of IMRT over conventional 2D and 3D RT techniques, the initial data are impressive in both locoregional disease control and reduced toxicities. A role for IMRT as a standard of care in the treatment of NPC is foreseeable. Aside from IMRT, other techniques are being explored in both early-stage and advanced NPC to improve upon locoregional response rates while limiting toxicities. Both high-dose-rate intracavitary brachytherapy and stereotactic radiosurgical boost have been shown to increase locoregional control rates when given as an adjuvant to external beam RT (Pai *et al*, 2002; Le *et al*, 2003; Lu *et al*, 2004). Given the preliminary data afforded by these phase II studies, the future direction of RT in the treatment of NPC will certainly be a move away from 2D RT to the adoption of 3D RT with IMRT, while a more defined role for stereotactic radio surgery and intracavitary brachytherapy in the management of locally advanced NPC still needs to be established.

## MOLECULAR TARGETED THERAPY

Recurrent or metastatic NPC remains largely an incurable disease, although there have been reports of long-term survivors among those who achieved complete responses to conventional chemotherapy (Fandi *et al*, 2000). Combinations of cytotoxic chemotherapeutic agents with antitumour activity, such as the platinums, 5-fluorouracil, methotrexate, anthracyclines, gemcitabine and taxanes, typically yield high response rates of limited duration, and are associated with normal tissue toxicity. At the Princess Margaret Hospital, gemcitabine-based therapy has been adopted as first-line therapy for recurrent or metastatic NPC, with response rates of 34%, as a single agent among platinum-refractory patients, or 64% when used in combination with cisplatin among platinum-sensitive patients. Median duration of response was 17 and 24 weeks, and 1-year survival rates were 48 and 69%, respectively (Ma *et al*, 2002) In order to maximise the proportion of long-term survivors among patients with recurrent or metastatic NPC, better systemic agents are needed to increase the likelihood of complete responses. With a potentially superior therapeutic index, molecular targeted agents represent exciting compounds, which may complement the use of conventional chemotherapy in this disease.

Several molecular targets have been identified in tumour specimens of patient with NPC. Expression or overexpression of the following receptors has been evaluated in NPC: epidermal growth factor receptor (EGFR), vascular endothelial growth factor (VEGF), c-KIT and c-erbB-2 (HER2). To date, no reports of mutational analyses of any of these molecular targets have been published.

A retrospective study was performed to assess the correlation between the expression of EGFR and treatment outcome in patients with advanced stage disease (Chua DT *et al*, 2004). All patients in this group were treated with induction chemotherapy followed by RT. Of the specimens tested, 89% of the cases demonstrated EGFR expression. The extent and intensity of the staining did not correlate with tumour burden or extent of disease. Patients in whom greater than 25% of their tissue cells stained positive for EGFR had a reduced survival and increased risk of locoregional failure compared to those patients who did not. A second trial specifically evaluated undifferentiated carcinoma of the nasopharynx (Leong *et al*, 2004). EGFR expression levels were ascertained in 75 patients diagnosed with undifferentiated NPC. Rates of overexpression were reported at 83%. In this cohort, the patients with EGFR overexpression did have a more locally aggressive disease, but DFS and OS rates did not alter significantly between the two groups. Given the finding of the high prevalence of EGFR overexpression, a phase II study of cetuximab (Erbitux, C-225) in combination with carboplatin for patients with metastatic or recurrent NPC was undertaken (Chan *et al*, 2003). Of 60 patients, 56 screened were positive for EGFR expression and enrolled in the trial. The overall response rate was 17%, and partial response or stable disease accounted for 66%. Based on this preliminary data, EGFR is a viable target for further clinical trials. Furthermore, a recent randomised phase III trial of RT with or without cetuximab in patients with locoregionally advanced non-nasopharyngeal squamous cell cancers of the head and neck has shown a significant survival benefit with the addition of cetuximab, and toxicity profile was acceptable. This trial has definite implications for the role of anti-EGFR agents in locally advanced NPC, and further evaluations are needed to determine how best to incorporate them into current standard chemoradiotherapy regimens (Bonner *et al*, 2004).

Vascular endothelial growth factor is an angiogenic factor, which contributes to angiogenesis and subsequent growth, invasion and metastasis of tumours. Expression of VEGF has been compared between tissue samples obtained from normal nasopharynx without tumour, nasopharyngeal benign tumours and NPC: the rates of VEGF expression were 10, 40 and 80%, respectively. As well, expression of VEGF in advanced disease was increased with a statistical significance compared with those in early-stage disease (Guang-Wu *et al*, 2000). The role of anti-VEGF therapy has not been explored, but could serve as a potential target in future studies.

A retrospective study of 49 patients with NPC assessed the overexpression of HER2 and c-KIT (Bar-Sela *et al*, 2003). c-KIT overexpression was found in 33% of the cases and was associated with a positive Ebstein–Barr virus (EBV) status (by EBER *in situ* hybridisation) in patients whose histology showed nonkeratinising or undifferentiated carcinoma. These cases were associated with a trend towards a better survival. Whether or not a potential therapeutic role for inhibitors of c-KIT such as imatinib mesylate exists remains unknown at the present. In this same cohort of patients, HER2 overexpression was uniformly negative. This result differs from a Hong Kong study of 78 Chinese patients with undifferentiated NPC (Ma *et al*, 2003), in which HER2 overexpression was observed in 31% of the patients and conferred an association with increased stage of disease. Lastly, a study from Guangzhou in Southern China performed both immunohistochemical and fluorescent *in situ* hybridisation (FISH) analyses of HER2 in 45 cases of NPC. While 33% of patients had HER2 expression in tumour samples by immunohistochemical staining, this did not correlate with clinical outcome, and no significant alterations in gene copy number of HER2 was detected by FISH (Yan *et al*, 2002). At the present, no studies of trastuzumab or other HER2 inhibitors have been reported in NPC.

## EBV IN NPC AND IMMUNOTHERAPY

A clear association exists between EBV infection and NPC. EBV is present in the cells of almost all primary and metastatic NPC, regardless of tumour histology, extent of disease or patient geographic location. It has been postulated that plasma EBV DNA quantification can be useful to follow patients and predict outcome of treatment (Lin *et al*, 2004). A recent study was conducted to investigate the significance of plasma EBV DNA concentrations in patients with advanced NPC. In all, 99 patients were treated with neodjuvant cisplatin and 5-fluorouracil given on alternate weeks for 10 weeks followed by RT therapy to a total of 70–74 Gy. Blood samples were obtained for measurement of EBV DNA concentrations using real-time quantitative polymerase chain reaction. Samples were obtained 1 day prior to beginning treatment, days 35 and 64 during neoadjuvant chemotherapy, and 1 week after completion of RT. Forty healthy volunteers, 20 patients cured of their disease for greater than 5 years and 19 patients with metastatic disease were used as controls. At baseline, 94 of 99 patients with stage III and IV disease, and all patients with metastatic disease had detectable plasma EBV DNA. No healthy controls had detectable EBV DNA. EBV DNA concentrations were significantly higher in the group that subsequently relapsed following therapy *vs* the group that remained in a clinical remission. Higher pretreatment EBV DNA values corresponded to a decreased OS and DFS. Based on this initial data, EBV DNA quantification seems promising as a marker to monitor and predict treatment outcomes in patients with advanced NPC.

Given this association of EBV and NPC, investigators have evaluated a role for immunotherapy. In-depth studies have established that NPC cells express two distinct EBV latent membrane proteins, LMP-1 and LMP-2. These proteins become attractive targets for adoptive immunotherapy. A phase I study of EBV-specific cytotoxic T lymphocytes (CTL) was undertaken in EBV-positive patients with advanced NPC. CTL were generated and then infused into 10 patients. Four patients treated in remission remained disease free 19–27 months after infusion. Of six patients with refractory disease, two had complete responses, one had a partial response, one had stable disease and two had no response (Straathof *et al*, 2004). Except for the development of increased swelling at the site of pre-existing disease in one patient, CTL were generally well tolerated by all patients. Based on the preliminary work, the administration of CTL is feasible and is associated with significant antitumour activity. Further studies utilising CTL should be continued.

## CONCLUSIONS

Based on the above data, a clear role for concomitant chemoradiotherapy followed by adjuvant chemotherapy has shown statistically significant improvement in OS and DFS for all histological types of locally advanced NPC. Neoadjuvant chemotherapy followed by concurrent chemoradiotherapy would be a reasonable variation, since the maintenance of chemotherapy dose intensity may be optimal using such a sequence. Further treatment questions arise in the management of local and distant failures. Intensity-modulated RT proves to be an improvement in RT delivery, and in several phase II studies has initially translated into better locoregional control and reduced toxicities. In cases of recurrent or distant disease, the standard of care is diverse.

Given the complex nature of this disease and the high risk for development of distant failures, new treatment regimens need to be developed for these patients. Exploration of the role of targeted agents such as inhibitors of EGFR, VEGF, c-KIT and HER2 are necessary. Adoptive immunotherapy with EBV-specific CTL awaits further exploration. Certainly, the treatment strategies for NPC will continue to change and evolve as a better understanding is gained of the molecular and immune mechanisms that drive this disease.

## Figures and Tables

**Table 1:** Randomised trials of chemotherapy with RT *vs* RT alone in locally advanced NPC

**Author and year**	**No. of pts.**	**Treatment arms**
*Neoadjuvant chemotherapy+RT vs RT alone*		
VUMCA (1996)	a: 171	a: B 15 mg D1, 12 mg m^−2^ d^−1^ D1–5, E 70 mg m^−2^ D1, and P 100 mg m^−2^ D1 every 21 days × 3 → RT
	b: 168	b: RT
Hareyama *et al* (2002)	a: 40	a: P 80 mg m^−2^ D1 and F 800 mg m^−2^ d^−1^ D2–5 every 21days × 2 → RT
	b: 40	b: RT
Chua *et al* (1998)	a: 167	a: P 60 mg m^−2^ D1 and E 110 mg m^−2^ D1 every 21 days × 2–3 → RT
	b: 167	b: RT
Ma *et al* (2001)	a: 224	a: P 100 mg m^−2^ D1, B 10 mg m^−2^ D1 and 5, and F 800 mg m^−2^ d^−1^ D1–5 every 21 days × 2–3 → RT
	b: 225	b: RT

*Concurrent chemotherapy+RT vs RT alone*		
Lin *et al* (2003)	a; 141	a: P 20 mg m^−2^ d^−1^ D1–4 and F 800 mg m^−2^ d^−1^ D1–4 on weeks 1, 5+RT
	b: 143	b: RT
Chan *et al* (2002, 2004)	a: 174	a: P 40 mg m^−2^ weekly+RT
	b: 176	b: RT

*Adjuvant chemotherapy+RT vs RT alone*		
Rossi *et al* (1988)	a: 113	a: RT → V 1.2 mg m^−2^ D1, C 200 mg m^−2^ d^−1^ D1–4, and A 40 mg m^−2^ D1 every 28 days × 6
	b: 116	b: RT
Chi *et al* (2002)	a: 77	a: RT → P 20 mg m^−2^ D1, F 2200 mg m^−2^ D1, and L 120 mg m^−2^ D1 weekly × 9
	b: 77	b: RT

*Neoadjuvant and adjuvant chemotherapy+RT vs RT alone*		
Chan *et al* (1995)	a: 37	a: P 100 mg m^−2^ D1 and F 1000 mg m^−2^ d^−1^ D2–4 every 21 days × 2 → RT → P 100 mg m^−2^ D1 and F 1000 mg m^−2^ d^−1^ D2–4 every 21 days × 4
	b: 40	b: RT

*Concurrent and adjuvant chemotherapy+RT vs RT alone*		
Al-Sarraf *et al* (1998, 2001)	a: 93	a: P 100 mg m^−2^ D1, 22, 43+RT → P 80 mg m^−2^ D1 and F 1000 mg m^−2^ d^−1^ D1–4 weeks 11, 15, 19
	b: 92	b: RT
Wee *et al* (2004)	a: 111	a: P 25 mg m^−2^ d^−1^ D1–4 weeks 1, 4, 7+RT → P 20 mg m^−2^ d^−1^ D1–4 and F 1000 mg m^−2^ d^−1^ D1–4 weeks 11, 15, 19
	b: 109	b: RT
Lee *et al* (2004)	a: 172	a: P 100 mg m^−2^ D1, 22, 43+RT → P 80 mg m^−2^ D1 and F 1000 mg m^−2^ d^−1^ D1–4 weeks 11, 15, 19
	b: 176	b: RT
Kwong *et al* (2004)	a_1_: 57	a_1_: UFT 600 mg d^−1^+RT → P 100 mg m^−2^ D1 and F 1000 mg m^−2^ d^−1^ D1–3 alternating with V 2 mg, B 30 mg, and M 150 mg m^−2^ every 21 days × 6
	a_2_: 53	a_2_: UFT 600 mg d^−1^+RT
	a_3_: 54	a_3_: RT → P 100 mg m^−2^ D1 and F 1000 mg m^−2^ d^−1^ D1–3 alternating with V 2 mg, B 30 mg, and M 150 mg m^−2^ every 21 days × 6
	b: 55	b: RT

NPC=nasopharyngeal carcinoma; RT=radiotherapy; P=cisplatin; F=fluorouracil; B=bleomycin; E=epirubicin; V=vincristine; C=cyclophosphamide; A=adriamycin; L=leucovorin; M=methotrexate; UFT=uracil–tegafur; a(a_1_, a_2_, a_3_)=combined therapy arm; b=radiotherapy alone arm; d=day; D=day; pts.=patients.

**Table 2:** OS and DFS of randomised trials of chemotherapy with RT *vs* RT alone in locally advanced NPC

**Author**	**Treatment arm**	**OS**	**DFS**	**Median F/U (mo)**
*Neoadjuvant chemotherapy+RT vs RT alone*				
VUMCA (1996)	a	3 yr – 60%	3 yr – 52% *P<0.01	49
	b	54%	32%	
Hareyama *et al* (2002)	a	5 yr – 60%	5 yr – 55%	49
	b	48%	43%	
Chua *et al* (1998)	a	3 yr – 78%	3 yr – 48%	30
	b	71%	42%	
Ma *et al* (2001)	a	5 yr – 63%	5 yr – 59% *P=0.05	62
	b	56%	49%	
Chua *et al* (2004)^a^	a	5 yr – 64% *P=0.029	5 yr – 51% *P=0.014	67
	b	58%	43%	

*Concurrent chemotherapy+RT vs RT alone*				
Lin *et al* (2003)	a	5 yr – 72% *P<0.01	5 yr – 72% *P<0.01	65
	b	54%	53%	
Chan *et al* (2002, 2004)	a	N/A	2 yr – 76%	33
	b	N/A	69%	
	a	5 yr – 70% *P<0.05	5 yr – 60%	65
	b	59%	52%	

*Adjuvant chemotherapy+RT vs RT alone*				
Rossi *et al* (1988)	a	5 yr – 55%	5 yr – 54%	49.5
	b	61%	50%	
Chi *et al* (2002)	a	4 yr – 59%	4 yr – 58%	43
	b	67%	56%	

*Neoadjuvant and adjuvant chemotherapy+RT vs RT alone*				
Chan *et al* (1995)	a	2 yr – 80%	2 yr – 68%	28.5
	b	81%	72%	

*Concurrent and adjuvant chemotherapy+RT vs RT alone*				
Al-Sarraf *et al* (1998, 2001)	a	3 yr – 76% *P<0.001	3 yr – 66% *P<0.001	32.4
	b	46%	26%	
	a	5 yr – 67% *P<0.001	5 yr – 58% *P<0.001	60
	b	37%	29%	
Wee *et al* (2004)	a	2 yr – 85% *P=0.02	2 yr – 76%	N/A
	b	77%	62%	
Lee *et al* (2004)	a	3 yr – 77%	3 yr – 67%	25
	b	76%	61%	
Kwong *et al* (2004)	a_1_	3 yr – 89%	3 yr – 70%	32.5
	a_2_	84%	69%	
	a_3_	71%	54%	
	b	83%	61%	

* Statistically significant result (*P*⩽0.05). a(a_1_, a_2_, a_3_)=combined therapy arm; b=radiotherapy alone arm; OS=overall survival; DFS=disease-free survival; NPC=nasopharyngeal carcinoma; RT=radiotherapy; N/A=not applicable; yr=year; mo=months.

a This reference is an updated analysis of patients from Chua *et al* (1998) and Ma *et al* (2001).

**Table 3:** Initial RT dose and schedule of randomised trials of chemotherapy with RT *vs* RT alone in locally advanced NPC

**Author**	**Weekly fractions**	**Primary tumour dose**	**Involved nodes dose**	**Remaining nodal area (Gy)**
*Neoadjuvant chemotherapy+RT vs RT alone*				
VUMCA (1996)	5 × 2 Gy d^−1^	65–70 Gy	65 Gy	50
Hareyama *et al* (2002)	5 × 2.0 Gy d^−1^ or4 × 2.2 Gy d^−1^	66–68 Gy	66–68 Gy	50
Chua *et al* (1998)	5 × 2 Gy d^−1^ (*n*=110) orhypofractionated (*n*=176)	66–70 Gy (36%)>70–74 Gy (64%)	60–66 Gy (82.5%)>66–76 Gy (17.5%)	60
Ma *et al* (2001)	5 × 2.0 Gy d^−1^	68–72 Gy	60–62 Gy	50

*Concurrent chemotherapy+RT vs RT alone*				
Lin *et al* (2003)	5 × 2.0 Gy d^−1^ (*n*=240) orhyperfractionated (*n*=44)	70–74 Gy	70–74 Gy	50–60
Chan *et al* (2002, 2004	5 × 2 Gy d^−1^	66 Gy	N/A	N/A

*Adjuvant chemotherapy+RT vs RT alone*				
Rossi *et al* (1988)	1.8 Gy d^−1^ (*n*=103) orhyperfractionated (*n*=13)	60–70 Gy	60–70 Gy	50
Chi *et al* (2002)	5 × 1.8–2.0 Gy d^−1^	70–72 Gy	70–72 Gy	50

*Neoadjuvant and adjuvant chemotherapy+RT vs RT alone*				
Chan *et al* (1995)	N/A	66 Gy±boost	58 Gy+7.5 Gy boost	58

*Concurrent and adjuvant chemotherapy+RT vs RT alone*				
Al-Sarraf *et al* (1998, 2001)	5 × 1.8–2.0 Gy d^−1^	70 Gy	66–70 Gy	50
Wee *et al* (2004)	2.0 Gy d^−1^	70 Gy	N/A	N/A
Lee *et al* (2004)	5 × 2.0 Gy d^−1^	66 Gy	66 Gy	N/A
Kwong *et al* (2004)	4 × 2.5 Gy d^−1^ to40 Gy then5 × 2.5 Gy d^−1^ or	62.5 Gy	62.5 Gy	N/A
	5 × 2.0 Gy d^−1^	68 Gy	66 Gy	N/A

Gy=Grays; d=day; N/A=not applicable; RT=radiotherapy; NPC=nasopharyngeal carcinoma; a(a_1_, a_2_, a_3_)=combined therapy arm, b=radiotherapy alone arm.
